# The Impact of Apical Patency in the Success of Endodontic Treatment of Necrotic Teeth with Apical Periodontitis: A Brief Review

**DOI:** 10.7508/iej.2016.01.012

**Published:** 2015-12-24

**Authors:** Ricardo Machado, Carlos Henrique Ferrari, Eduardo Back, Daniel Comparin, Luiz Fernando Tomazinho, Luiz Pascoal Vansan

**Affiliations:** a *Department of Multidisciplinary Clinic I and II and Supervised Stage in Multidisciplinary Clinic I (Endodontics), Paranaense University-UNIPAR, Francisco Beltrão, Paraná, Brazil; *; b * São Jose dos Campos Dental School, State University of São Paulo-UNESP, São Jose dos Campos, São Paulo, Brazil;*; c *Clinical Practice, Joinvile, Santa Catarina, Brazil; *; d *Clinical Practice, Cunha Porã, Santa Catarina, Brazil; *; e *Department of Multidisciplinary Clinic I and II and Supervised Stage in Multidisciplinary Clinic II (Endodontics), Paranaense University-UNIPAR, Francisco Beltrão, Paraná, Brazil;*; f * Department of Endodontics, Ribeirão Preto Dental School, University of São Paulo, Ribeirão Preto, São Paulo, Brazil*

**Keywords:** Apical Patency, Endodontic Success, Periradicular Disease, Root Canal Therapy

## Abstract

Accumulation of soft tissue or dentinal remnants in the apical region is a common event that can cause blockage of root canals. This event can be avoided if apical patency is performed during the root canal shaping procedures. However, there is no consensus on the role of apical patency in relation to the success of endodontic treatment of necrotic teeth with apical periodontitis. Therefore, the purpose of this paper was to conduct a brief review on the role of apical patency in guaranteeing the success of endodontic treatments of necrotic teeth with apical periodontitis considering two other key points; the root canal anatomy and microbiology.

## Introduction

Blockage of the root canal in the apical region by remnants of dental hard and soft tissue debris, may cause procedural errors such as apical transportations, ledge and perforation. These debris may also contain bacteria capable of maintaining or inducing periradicular disease [1-4]. For these reasons, apical patency has been suggested [5-7]. 

The most common method for performing this procedure is to use a so-called patency file during root canal instrumentation. This file can be defined as a small flexible K-file, which is moved passively through the apical foramen without widening it [5]. In addition to avoiding the procedural errors referred above, apical patency minimizes the risk of losing working length, enhances irrigation and improves the tactile sensation of the clinician [5, 8-11]. On the other hand, according to many others studies, apical patency can promote extrusion of contaminated debris and subsequently irritate periradicular tissues [2, 12-16]. 

Whereas most studies are merely speculative regarding the advantages and disadvantages of performing or not apical patency [7-9], the purpose of this paper was to conduct a brief review of the literature about the role of this procedure in guaranteeing the success of endodontic treatments in necrotic teeth with apical periodontitis.

***Anatomical considerations***

An unchangeable high-impact factor that remains in endodontics is the root canal anatomy. The complexity of this factor has been verified by several methods over the years such as diaphonization, microscopic investigation, radiographic methods, scanning electron microscopy (SEM), cone-beam computed tomography (CBCT) and computed micro-tomography (micro-CT) [17-26]. This complexity is considerably higher in the apical third, regardless of the tooth type and includes apical deltas and lateral canals [22, 25, 27-30].

Morfis *et al*. [22] found no principal foramen in 24% of maxillary premolars and in 26% of maxillary incisors. In a study on the mesiobuccal roots of maxillary molars, Verma and Love [28] found a 20% presence of two foramina and a 65% presence of three or more apical foramina. A study performed by Meder-Cowherd *et al.* [25] on the palatal roots of maxillary molars found a high variation of apical anatomy with apical delta in 12% of the specimens. Therefore, sometimes it is clinically impossible to reach the apical foramen, despite all the current technological advances [29, 30]. However, according to literature, there are no robust scientific evidences showing that these cases are necessarily doomed to fail. Also, achieving apical patency does not ensure the success of the treatment [31]. Even in cases where apical patency is obtained, there are other anatomical variations, such as apical deltas, lateral canals and multiple foramina, which can harbor bacteria with a potential to induce or maintain a periradicular disease [21, 22, 25, 27-32].

***Microbiological*** ***considerations***

The endodontic treatment of vital teeth is essentially a prophylactic treatment, because the pulp space is usually free of microorganisms, and the prevailing rationale for treatment is to prevent probable infection and consequent periradicular disease [33-39]. On the other hand, in cases of necrotic pulps, intra-radicular infection is already established, and endodontic procedure should focus not only on preventing new microorganisms from being introduced into the root canal system, but also on reducing those located there in [40-50]. 

Persistence of bacteria can influence the outcome of the endodontic treatment in five possible ways: *i)* when they are able to survive without nutrients; *ii)* when they resist treatment-induced disturbances in the ecology of the bacterial community, (including disruption of quorum-sensing systems, food webs/chains and genetic exchanges, and disorganization of protective biofilm structures); *iii)* when they reach a climax population density (load) high enough to inflict damage on the host; *iv)* when they possess attributes of virulence expressed in the modified environment and reaching concentrations strong enough to directly or indirectly induce damage to the periradicular tissues and *v)* when they have unrestrained access to periradicular tissues through apical/lateral foramina [49]. 

Specifically related to the last factor mentioned above, apical patency cannot provide any advantage, because there is no real possibility of reaching microorganisms in lateral canals and apical deltas using the techniques currently known for this procedure.

***Can apical periodontitis heal after endodontic treatments without performing apical patency?***

Many investigators have retrospectively evaluated the influence of various factors that may affect the outcome of root canal treatments or retreatments [51-54]. In these studies, the limits of apical filling appear regularly as one of these factors. However, in most of these studies, the limits were measured based only on post-operative radiographs. Very likely, several cases had inaccessible root canals (without apical patency), even when the apical filling limits were observed to be within 2-mm distance from the apex. 

Apical patency is not always achieved. Severe curvatures, broken instruments, miscalculation of canal length, development of ledges, obliteration and the anatomy of the root canals are among the most common reasons [55-64]. However, not all of these cases are associated with failure. Considering that the failure cannot be solely based on the absence of apical patency, there are several important factors that should not be underestimated.

Failure of nonsurgical root canal treatment or retreatment is usually related to residual bacteria (persistent infection) or reinfection of an already disinfected root canal environment (secondary infection) [11, 31, 65-70]. For surviving bacteria to maintain or induce a periradicular disease, they must adapt to the new environment represented by the filled canal, have a steady source of nutrients, have available space to multiply, and reach numbers high enough to elicit tissue damage [31, 46, 49, 65-67, 69, 70].

Therefore, the postulation of periradicular disease perpetuation simply because apical patency was not obtained, is a very questionable inference. This statement is mainly based on the following considerations: *a)* a high bacterial load diminishes substantially after correct instrumentation of the cervical and middle thirds; *b)* in several situations, even when apical patency is not achieved using files, *chemical apical patency *may occur using irrigating solutions, medications and sealers that are able to "disrupt microbiologically" this region; and *c)* only the main canal can be subjected to the effective action of apical patency files, whereas a large number of lateral and smaller canals cannot be reached. The endodontic literature confirms these statements, whereas most cases of endodontic failure, evidenced by histological findings, are associated with microorganisms in lateral canals and apical deltas and/or with extra-radicular biofilms [31, 67, 69].

## Conclusion

This paper demonstrated that apical patency may not be strictly necessary However, there are no robust clinical or scientific evidences showing a direct correlation between apical patency and success of endodontic treatments of necrotic teeth with apical periodontitis.
